# Association between Plasma Trace Element Concentrations in Early Pregnancy and Gestational Diabetes Mellitus in Shanghai, China

**DOI:** 10.3390/nu15010115

**Published:** 2022-12-27

**Authors:** Ting Wu, Tao Li, Chen Zhang, Hefeng Huang, Yanting Wu

**Affiliations:** 1 The International Peace Maternity and Child Health Hospital, School of Medicine, Shanghai Jiao Tong University, Shanghai 200030, China; 2 Shanghai Key Laboratory of Embryo Original Diseases, Shanghai 200030, China; 3 Obstetrics and Gynecology Hospital, Institute of Reproduction and Development, Fudan University, Shanghai 200030, China; 4 Research Units of Embryo Original Diseases, Chinese Academy of Medical Sciences, Shanghai 200030, China; 5 Women’s Hospital, School of Medicine, The Key Laboratory of Reproductive Genetics, Ministry of Education (Zhejiang University), Hangzhou 310058, China

**Keywords:** gestational diabetes mellitus, nickle, trace elements, restricted cubic spline, LASSO regression, quantile g-computation, BKMR models

## Abstract

(1) Background: Trace elements play important roles in gestational diabetes mellitus (GDM), but the results from reported studies are inconsistent. This study aimed to examine the association between maternal exposure to V, Cr, Mn, Co, Ni, and Se in early pregnancy and GDM. (2) Methods: A nested case-control study with 403 GDM patients and 763 controls was conducted. Trace elements were measured using inductively coupled plasma-mass spectrometry in plasma collected from pregnant women in the first trimester of gestation. We used several statistical methods to explore the association between element exposure and GDM risk. (3) Results: Plasma V and Ni were associated with increased and decreased risk of GDM, respectively, in the single-element model. V and Mn were found to be positively, and Ni was found to be negatively associated with GDM risk in the multi-element model. Mn may be the main contributor to GDM risk and Ni the main protective factor against GDM risk in the quantile g computation (QGC). 6.89 μg/L~30.88 μg/L plasma Ni was identified as a safe window for decreased risk of GDM. (4) Conclusions: V was positively associated with GDM risk, while Ni was negatively associated. Ni has dual effects on GDM risk.

## 1. Introduction

Gestational diabetes mellitus (GDM), which refers to diabetes diagnosed for the first-time during pregnancy, is one of the most common medical complications of pregnancy [1]. It is associated with substantial short- and long-term adverse complications for both mother and child. The documented prevalence of GDM varies substantially worldwide, ranging from 1% to >30% [2]. The incidence rate of GDM has been increasing worldwide and is approximately 14.8% (95% CI 12.8, 16.7%) in China according to the latest meta-analysis involving 79,064 Chinese participants [3]. In addition to traditional risk factors, such as advanced maternal age, ethnicity, a previous history of gestational diabetes, and a family history of type 2 diabetes mellitus (T2DM), trace elements may play important roles in the development of diabetes [4].

Certain trace elements, such as chromium (Cr), have been suggested to participate in increasing insulin binding and insulin receptor number [5]. Vanadium (V) was found to participate in inhibiting glucose release, improving gluconeogenesis-related enzyme activity, and exerting an insulin-sensitizing effect [6]. Meanwhile, some essential elements, such as manganese (Mn) were found to be associated with a higher risk of hyperglycemia by inhibiting glucose-stimulated insulin secretion and inducing inflammation and oxidative stress [7]. However, not all human studies support the results from laboratory studies. An adult cohort study from Southern Spain suggested that concentrations of certain trace elements (such as Cr) in adipose tissue are associated with the risk of incident T2DM, while V might have a protective effect [8]. A case-control study in China indicated that higher levels of serum selenium (Se) were associated with increased T2DM risk [9].

Some trace elements have recently been suggested to be associated with the risk of GDM in epidemiologic studies. A prospective study demonstrated that increased concentrations of urinary nickel (Ni), Cobalt (Co), and V in early pregnancy are associated with an elevated risk of GDM [10]. In contrast to the results of the above research, two case-control studies indicated an inverse association of V exposure with GDM [11,12], which was reflected by plasma V concentrations and meconium V concentrations. No significant association was found between blood Ni and GDM in the single-metal model in a Chinese birth cohort study [13]. Moreover, a nested case-control study in Xiamen, China, measured Cr concentrations in meconium from newborns delivered by mothers with GDM (137 cases) and without GDM and found a positive association between Cr concentration and GDM prevalence in a dose-dependent manner [14]. One recent meta-analysis showed that the serum Se level of patients with GDM was lower than that in healthy pregnant women. However, no association was found between plasma Se, Cr, and GDM in another nested case-control study [15]. A higher concentration of Mn within a certain range before 24 weeks gestation was demonstrated to impair fasting plasma glucose during pregnancy in a retrospective study [16]. Additionally, a French mother-child cohort study did not find a significant association between blood Mn and the prevalence of GDM [17].

Thus far, the results and conclusions on the relationship between the six trace elements—V, Cr, Mn, Co, Ni, and Se—and GDM are limited and contradictory. In addition, it is essential to study the joint effects of trace elements on GDM risk because elements in the environment exist in the form of co-exposure, and the specific elements included in the analysis individually could be potentially confounded by other elements to which pregnant women are also exposed from the same source. However, when exploring the effects of a multielement mixture in a traditional way, highly unstable results may be obtained if incorporating two or more highly correlated (collinear) elements in a regression model [18]. In recent years, various interdisciplinary methods [19] have been developed to address such issues.

In the present study, we aimed to explore the relationship between these six plasma trace element concentrations before 14 gestational weeks and the risk of GDM. We used least absolute shrinkage and selection operator (LASSO) regression, quantile g computation (QGC), and Bayesian Kernel Machine Regression (BKMR) to screen out independent variables, assess the joint effect of elements on GDM risk and determine the contribution of each element on GDM risk, restricted cubic spline (RCS) was employed to explore the dose-response relationship between elements exposure and GDM risk, with the hope to provide new insights for the prevention of GDM.

## 2. Materials and Methods

### 2.1. Study Population

This case-control study was nested in a prospective study initiated in Shanghai, China. From November 2020 to February 2021, pregnant women who visited the International Peace Maternal and Child Hospital (IPMCH) for the first prenatal examination between 8 and 14 gestational weeks and provided enough blood samples were included in the study (*n* = 2069).

The excluded participants were those: (1) who had multiple births (*n* = 52); (2) who were diagnosed with T2DM and other metabolic diseases before pregnancy (*n*= 31); (3) who had serious medical diseases such as cancer (*n* = 12); and (4) who had missing information on birth outcomes (*n* = 292) and missing blood samples (*n* = 92).

Among 1724 finally included pregnant women, 403 pregnant women were diagnosed with GDM and included in the GDM group, and a total of 763 controls were randomly selected from the remaining participants by maternal pre-pregnancy BMI and maternal age (case/control = 1:2 for 360 cases and case/control = 1:1 for 43 cases).

All of the participants in the study signed informed consent forms. This study was approved by the ethics committee of the IPMCH.

### 2.2. Data Collection

Baseline information was obtained from electronic medical records, including maternal age, ethnic group, pre-pregnancy body mass index (BMI), reproductive history, family and personal disease history, smoking exposure, alcohol consumption, education levels, household income, delivery method, and fetal sex. Maternal BMI was calculated using the formula BMI = weight (kg)/height (m^2^). Gestational age was calculated based on the gestational week of delivery and the first day of the last menstrual period. In the present study, smoking exposure was defined as positive if the mother had a smoking history, and alcohol consumption was considered positive if the mother had a drinking history.

### 2.3. Laboratory Measurements

Plasma concentrations of total cholesterol (CHOL), triglycerides (TG), high-density lipoprotein cholesterol (HDL), low-density lipoprotein cholesterol (LDL), apolipoprotein-A (APO-A), apolipoprotein-B (APO-B) and fasting plasma insulin (FPI) were measured by an automatic chemistry analyzer (BeckmanDXI800, Beckman, Bria, CA, USA). The homeostasis model of assessment-insulin resistance (HOMA-IR) score was obtained according to the following formula: HOMA-IR = FPG (mmol/L) × FPI (µU/mL)/22.5.

Inductively coupled plasma-mass spectrometry (ICP-MS) was used for the determination of the six trace elements. ICP-MS is a quadrupole mass spectrometer, consists of basic components, including the peristaltic pump, nebulizer, spray chamber, ICP torch, interface cones, ion optics, quadrupole, and detector. It has been considered the gold standard analytical method for element measurements in biological samples which meet the interference elimination of the determination of different elements in the sample. We used the NexION 300X device (PerkinElmer, Waltham, MA, USA) and the stander mode for the measurements [20]. Blood was collected between 8 and 14 gestational weeks, and plasma was collected in EDTA tubes after centrifugation at 2000 rpm for 20 min. All plasma samples were frozen at −80 °C for storage and transferred to a 4 °C refrigerator the night before detection. Several standard curves were prepared by diluting the element standard solution (PerkinElmer, Waltham, MA, USA), and the value of the limit of detection (LOD) of each element was calculated. Plasma (100 μL) was diluted 20 times with sample diluent (1% TMAH1 + % nitric acid) and fully vibrated before detection. See Table S1 for the LOD and detection rate of each element. When the plasma element concentration was below the LOD, LOD/√2 was used instead. Standard samples were detected in each batch (30 samples) for quality control purposes.

### 2.4. Diagnosis of GDM

At 24–28 weeks of gestation, an oral glucose tolerance test (OGTT) was implemented by a 75 g glucose challenge. A diagnosis of GDM was made if fasting plasma glucose was ≥5.1 mmol/L (≥92 mg/dL), 1-h plasma glucose was ≥10.0 mmol/L (≥180 mg/dL), or 2-h plasma was ≥8.5 mmol/L (≥153 mg/dL), according to the recommendations from the Diabetes and Pregnancy Study Group (IADPSG) [21].

### 2.5. Statistical Analysis

The control group was matched for the GDM group by maternal age and pre-pregnancy BMI using the propensity score matching method (PSM) [22]. Basic demographic characteristics, plasma microelement concentrations, and clinical indicators of the study population were represented using N (%) for categorical variables and median and interquartile range (IQR) for continuous variables. Comparison between case and control groups was determined by the Wilcoxon rank sum test (for continuous variables) or Chi-square (χ2) test (for categorical variables). The concentrations of trace elements were natural log-transformed [Ln(X)] to normalize their distribution. The pairwise correlations among multiple elements were calculated by Spearman’s rank correlation analysis and a correlation-matrix heatmap was plotted. Conditional logistic regression was adopted to evaluate the association between the concentration of trace elements and the risk of GDM by odds ratios (ORs) and 95% confidence intervals (CIs). We chose covariates based on the literature review, stepwise regression, best subset selection, and biological reliability. Potential confounding factors and factors with significant differences between the case and control groups in univariate analysis were included in Model 4, including age (continuous variable), pre-pregnancy BMI (<18.5, 18.5–24, >24), family history of diabetes (yes or no), education level (<10, 10–12, ≥13 years), ethnic groups (Ethnic Han or others), household income level (<0.1 million, 0.2–0.3 million, >0.3 million), TG (continuous variable), CHOL (continuous variable), LDL-cholesterol (continuous variable), HDL-cholesterol (continuous variable) and APOB (continuous variable). Covariates screened by stepwise regression were included in Model 2, including education level, ethnic groups, TG, LDL-cholesterol, HDL-cholesterol, and APOB. Covariates including family history of diabetes, education level, ethnic groups, TG, LDL-cholesterol, and APOB, which were screened by best subset selection, were included in Model 3. The potential nonlinearity of the association of plasma trace elements with odds of GDM, OGTT value, and FPI was further examined using RCS with three knots at the 25th, 50th, and 75th percentiles of Ln (plasma element concentrations) assessed via R version 4.2.0 software (“rms” package).

LASSO regression, QGC, and BKMR models were used to screen out independent variables, assess the joint effect of elements on GDM risk, and determine the contribution of each element to GDM risk. In these analyses, we adjusted for the same variables as in Model 3 of the conditional logistic regression analysis. The 11 covariates and six elements were included in the LASSO regression, and the independent variables with greater influence on the dependent variable were screened when the regression coefficient was compressed to zero. These selected elements were included simultaneously in the multiple-element model adjusted or not adjusted for covariates selected by LASSO (family history of diabetes, education level, ethnic groups, household income level, TG, LDL-cholesterol, HDL-cholesterol, APOB). Quantile g computation, an adaptive adaptation modeling method with weighted quantile sum regression, was used to evaluate the different directions of mixed effects for individual elements and rank important constituents [23]. QGC was conducted using R version 4.2.0 with the “qgcomp” package. BKMR [24] was also used to assess the joint effect of all elements on the risk of GDM and the effect of an individual element as part of the element mixture via the R version 4.2.0 software (“bkmr” package). A PIP (prosterior inclusion probabilities) threshold of 0.5 was considered to be relatively important for individual element exposure to GDM risk.

All statistical analyses were performed using the SPSS 26.0 and R version 4.2.0 software. A *p*-value (two-tailed) < 0.05 was considered significant.

## 3. Results

### 3.1. Characteristics of the Study Population

The characteristics of the study population are presented in Table 1. The median age of the included pregnant women was 32 years. The study population was well-educated, with around 71.78% of educational level reaching university and higher, and the women who developed GDM were less educated than the women in the control group.

### 3.2. Levels of Plasma Trace Elements and Glucose and Lipid Metabolism Indices

The exposure levels of the six trace elements in the case and control groups are summarized in Table 2. There were significantly increased levels of plasma V in the GDM group but significantly lower plasma concentrations of Cr and Se. Correlations between trace elements ranged from 0.07–0.82 in Spearman’s rank correlation analysis (Figure S1). As shown in Table S2, despite Apo-A, other glucose and lipid metabolism indices were significantly different between the case and control groups, with FPG, OGTT-1h, OGTT-2h, FPI, HOMA-IR, CHOL, TG, LDL-cholesterol, and Apo-B increased significantly and HDL-cholesterol decreased significantly in the GDM group.

### 3.3. Association between Plasma Trace Elements and Risk of GDM

The results of the conditional logistic regression are shown in Table 3. The plasma level of V was positively associated with the risk of GDM, and every unit increase in the natural log of V exposure was associated with 39% (OR = 1.39 (95% CI 1.14, 1.69)) a higher risk of GDM. In contrast, the concentration of plasma Ni was negatively associated with the risk of GDM, and every unit increase in the natural log of Ni exposure was associated with 14% (OR = 0.86 (95% CI 0.77, 0.97)) a lower risk of GDM. Elevated plasma concentrations of Cr, Mn, Co, and Se were not associated with the risk of GDM.

### 3.4. Dose-Response Association of Plasma Trace Element Exposure with GDM Risk, Glucose, and Insulin Level

**Figure 1:**
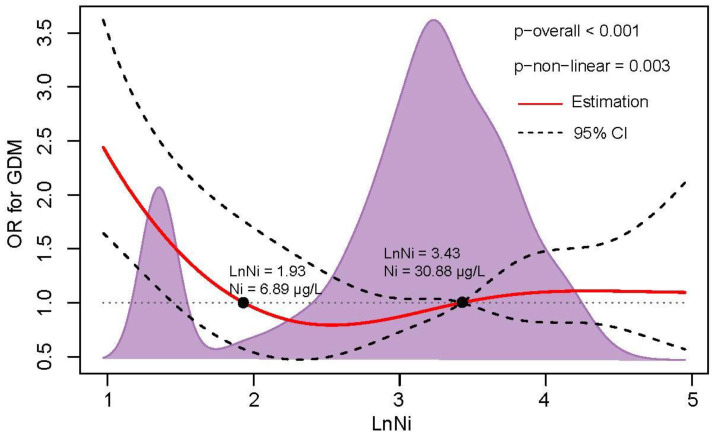
The dose-response relationship of plasma Ni level with GDM risk. RCS regression was used to analyze the dose-response relationship of Ln-Ni with GDM risk after adjusting for family history of diabetes, education level, ethnic groups, TG, LDL-cholesterol, and APO-B. The knots were located at the 25th, 50th, and 75th percentiles. The red line and black dotted line represent the OR value and 95% CI, respectively. The black points represent OR = 1, and the corresponding value of plasma Ni concentration is presented.

The potential nonlinearity of the relation between Ln- Ni (*p* overall < 0.001, *p* nonlinearity = 0.003) and the risk of GDM was observed in the restricted cubic spline model (Figure 1). In the relatively low levels (<6.89 μg/L) and higher levels (>30.88 μg/L) of plasma Ni, a positive correlation was found between plasma Ni and GDM risk. U-shaped exposure relationships were observed between Ni and FPI (*p* = 0.038), FPG (*p* = 0.006), OGTT-1h (*p* < 0.001), and OGTT-2h (*p* = 0.036) (Figure S2). Additionally, positive dose-response relationships were also observed between V and FPI (*p* = 0.005) and FPG (*p* = 0.004) (Figure S3), Mn and FPI (*p* = 0.018) (Figure S4), and Co and FPI (*p* = 0.039), OGTT-1h (*p* = 0.024) and OGTT-2h (*p* = 0.044) (Figure S5). Nonlinear relationships were not observed between Cr and Se and glucose or insulin levels (Figures S6 and S7).

### 3.5. Associations of Metallic Elements Screened by LASSO Regression and Their Coexposure with GDM Risk

The results of LASSO regression showed that all six trace elements had a strong effect on GDM risk (Figure S8). Next, we fitted a logistic regression model and brought all six elements into the model, and additionally adjusted covariates selected by the LASSO regression (Table 3). In this part, increasing Ln-V (OR = 1.27 (95% CI 1.01, 1.60)) and Ln-Ni (OR = 0.72 (95% CI 0.60, 0.86)) were positively and negatively related to the increased risk of GDM, respectively. In addition, increasing Ln-Mn was also observed to be positively associated with an increased risk of GDM (OR = 1.70 (95% CI 1.22, 2.36)).

### 3.6. Quantile G-Computation Analyses

**Figure 2:**
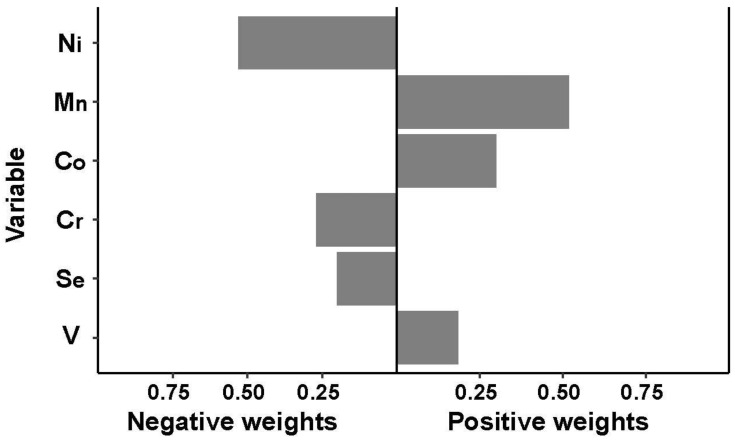
Association between trace element levels and GDM based on quantile g-computation analyses. The estimated weights of each element in the mixture were presented by bootstrapping in either direction.

QGC analysis showed that increasing the Ln-element mixture by one unit was not associated with an increased risk of GDM (OR = 0.97, 95% CI: 0.84, 1.13). The individual weights for each trace element mixture component are shown in Figure 2: Mn (59.1%), Co (31.6%), and V (9.3%) had a positive contribution, and Ni (61.5%), Cr (20.8%) and Se (17.7%) had a negative contribution.

### 3.7. Bayesian Kernel Machine Regression Analyses

**Figure 3:**
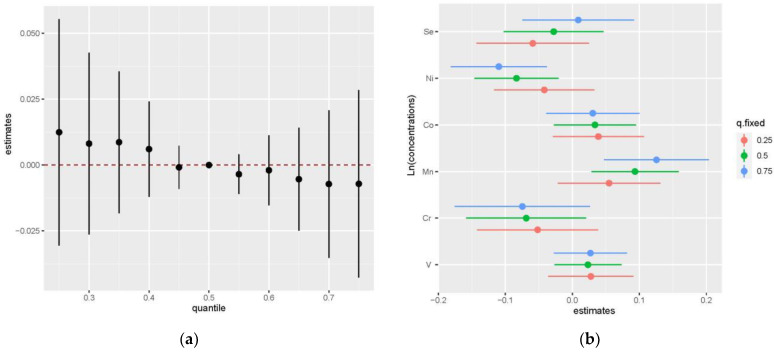
The joint effect of the trace element mixture on GDM by the BKMR model (**a**) The overall effects of element mixtures (estimates and 95% CI) in elements fixed to different percentiles compared to when they were at their medians (P50). (**b**) The effects of single exposure when an individual element was at its 75th percentile compared to when that exposure was at its 25th percentile, where all other exposures were fixed to a particular quantile (P25, P50, and P75). The results were adjusted by family history of diabetes, education level, ethnic groups, TG, and LDL-cholesterol.

The PIP of elements varied between 0.7 and 1 (V: 0.8666, Cr: 0.7746, Mn: 1.0000, Co: 0.7742, Se: 1.0000, Ni: 0.8062), and thus, all the elements could be considered important. The univariate relationship between each element and GDM risk is shown in Figure S8. Positive trends were observed between V, Co, and GDM risk (Figure S9a,d), and Cr showed a negative relationship with GDM risk (Figure S9b). A J-shaped relationship between Ni and GDM risk (Figure S9e) and an inverted J-shaped relationship between Mn, Se, and GDM risk (Figure S9c,f) were observed. Considering the comparable positive weight and negative weight of elements on GDM risk, the cumulative effect was not statistically significant, as shown in Figure 3a. Figure 3b illustrates the estimated contribution of individual exposures to the cumulative effect by comparing the GDM risk when a single element was at the 75th percentile compared to when it was at its 25th percentile, where all of the remaining elements were fixed to a particular quantile, such as P25, P50, and P75. When the other element percentiles increased, the higher percentiles of Ni and Mn (P75) presented a more significant negative effect and positive effect on GDM risk compared to their lower concentrations (P25), respectively. The trends of each element in the bivariate exposure-response analysis (Figure S10) were consistent with the univariate relationship analysis, except for the effect of Se on the GDM risk, which was inversely changed when Mn was at a high concentration (P75).

## 4. Discussion

In this study, we found that a higher level of plasma V and a lower level of plasma Ni before 14 weeks of pregnancy may be prospectively related to a higher risk of GDM in both single- and multiple-element models. Plasma Mn was found to be positively associated with an increased risk of GDM only in the multiple-element model, and there was no significant relationship between Cr, Co, and Se in the conditional logistic regression. A J-shaped relationship between plasma Ni concentrations and GDM and a U-shaped exposure-response relationship between plasma Ni and OGTT values and FPI were found in RCS analysis. The joint effect of element mixtures on the risk of GDM was not observed in the QGC analysis or the BKMR model. The results from QGC analysis indicate that Mn (59.1%), Co (31.6%), and V (9.3%) had a positive contribution, and Ni (61.5%), Cr (20.8%), and Se (17.7%) had a negative contribution to the risk of GDM. The association of incident GDM with V, Ni, and Mn was consistent in the outcomes of conditional logistic regression, QGC analysis, and BKMR analyses.

V was found to participate in inhibiting glucose release, improving gluconeogenesis-related enzyme activity, and exerting an insulin-sensitizing effect [6]. Two case-control studies showed a positive association between V in plasma [25] and serum [9] with diabetes risk. Two case-control studies based on pregnant women also reported that V exposure, reflected by meconium [12] and plasma V [11] concentrations, respectively, was inversely associated with the odds of GDM. Therefore, we expected that V would reduce the risk for GDM in our study, but the results indicated the opposite. The results from a prospective cohort study conducted in Wuhan, China, were close to those of our study, where there was a significant and positive association between urinary V and GDM based on single-metal models (OR = 1.28, 95% CI: 1.05–1.55) [10]. However, the biological sample of the abovementioned study was inconsistent with our study.

Notably, the median value of plasma V in the present study (6.25 μg/L) was much higher than that in previous studies (plasma V: 0.191 μg/L [25], 0.73 μg/L in GDM cases and 0.80 μg/L in controls [11]), which may account for the inconsistent result. Although V has been found to exert a beneficial effect on the metabolism of carbohydrates, the effective therapeutic dose is difficult to establish, and excess concentrations may lead to several toxic effects [5]. In addition, the health hazards of V, especially when it is at the highest oxidation state (+5), cannot be ignored. V can act as a strong pro-oxidant and pro-apoptotic factor, damage the antioxidant barrier, exacerbate lipid peroxidation (LPO), and lead to programmed cell death (apoptosis) [26]. Therefore, more research is warranted to further explore the effect of V on the risk of GDM and determine the safe exposure range of V.

The epidemiological evidence of Ni in glucose metabolism is limited and inconsistent, although Ni was suggested to adversely affect glucose metabolism by inducing hyperglycemia and glycogenolysis in laboratory studies [27]. Two studies based on Chinese adults and U.S. adults showed that increased urinary Ni concentration is associated with an elevated prevalence of diabetes [28,29]. However, a multisite and multiethnic cohort study of midlife women did not find an association between urinary Ni and an elevated risk of diabetes in midlife women [30]. Another nested case-control study obtained a similar result, and no significant associations were found between plasma Ni and incident diabetes [31]. The evidence of an association between Ni exposure and diabetes in pregnant women was insufficient, and no significant association was found between blood Ni and GDM in the single-metal model in a Chinese birth cohort study [13]. Nevertheless, another Chinese cohort study demonstrated that increased concentrations of urinary Ni in early pregnancy are associated with an elevated risk of GDM [10]. In the present study, a relationship between elevated maternal plasma concentrations of Ni and decreased risk of GDM was observed when evaluated individually or as an element mixture. Additionally, a J-shaped exposure relationship of Ln-Ni with the OR of GDM and U-shaped exposure relationships between Ln-Ni and the three OGTT values and FPI were all observed in the RCS analysis.

When interpreting our study results, the different study biological materials and the much higher concentration of Ni measured in our study should be considered (median (IQR) 30.67 (17.34–48.58) μg/L) when compared to previously published studies (median ranged from 2.48 to 6.484 μg/L [13,31,32]). Urine was a more commonly used biological material in previous studies because of the short half-life period of Ni. Considering that the main exposure source of Ni (from drinking water and food) may be stable in the pregnancy period, our findings may provide new insight into the effect of Ni on glucose metabolism. To the best of our knowledge, this is the first study to demonstrate the dual effect of plasma Ni exposure on the risk of GDM and provide a safety window value of Ni exposure (6.89 μg/L~30.88 μg/L) with potential clinical significance. Interestingly, the cutoff value of 30.88 μg/L was close to the median value of the study population (30.67 μg/L). Thus, we still recommend low levels of Ni exposure in daily life because Ni is potentially essential to the human body, but at high doses is toxic. More research is warranted to further verify our findings and explore the underlying mechanism.

Mn is both an essential nutrient and a potential toxicant, depending on the level of exposure. Mn supplementation may protect mitochondria and islets from ROS by enhancing MnSOD activity and protecting against diabetes [33], but it was also suggested that Mn can inhibit glucose-stimulated insulin secretion in β-cells by impairing mitochondrial function [7]. A cross-sectional study based on coke oven workers indicated that urinary Mn levels were positively associated with hyperglycemia but not with diabetes risk [34]. A U-shaped association between plasma manganese and T2DM was reported by a case-control study [35]. Nevertheless, no significant association was observed between second-trimester blood Mn and GDM in a French mother-child cohort study [17]. A retrospective cohort study from South China demonstrated that serum Mn may prospectively increase the late second trimester OGTT0 but not GDM risk [16].

We measured a relatively low concentration of Mn [median (IQR) 5.79 (3.51–8.90) μg/L] compared with other studies (median ranged from 6.52–21.85 μg/L) [15,17,35]. We found no significant association between Mn and GDM in the single-element model, but interestingly, when we included all the elements in the conditional logistic model, Mn showed a significant positive association with GDM. Additionally, Mn was positively associated with FPI level and was found to be the greatest contributor (59.1%) to GDM in the QGC analysis, which was similar to the results of QGC analysis from a large Japanese study (Mn: 47.4%) [36]. A similar result can also be observed in the BMKR model; when other element percentiles increased, Mn showed a more obvious positive association with GDM. We can speculate that Mn can promote the development of GDM through interactions with other elements such as Se as indicated in the bivariate exposure-response analysis of BKMR models but more evidence is needed to validate the speculation.

Cr, Co, and Se are essential trace elements in the human body. Cr was found to play a significant role in glucose metabolism and have beneficial effects on insulin sensitivity and lipid parameters [37]. Co is an important component of vitamin B12, and Se plays a critical role mainly as a selenoprotein.

Nevertheless, the role of Cr and Co in the development of diabetes mellitus in human studies remains controversial. A positive association was reported between Cr in adipose tissue with T2DM in a 16-year follow-up period prospective adult cohort study [8] and between Co in urine with T2DM in a study based on the National Health and Nutrition Examination Survey (NHANES, 1999–2010) [38]. In a case-control study involving 1471 patients with newly diagnosed T2DM, 682 individuals with newly diagnosed pre-DM indicated that plasma Cr concentrations were inversely associated with T2DM and pre-DM [39]. A negative linear relationship between urinary Co and FPG was found in an ongoing occupational cohort study in China [40]. A large case-control study elucidated a U-shaped relationship between plasma Co concentrations and newly diagnosed T2DM [41].

For pregnant women, Cr in meconium was found to be positively associated with GDM prevalence in a dose-dependent manner in a nested case-control study [14], while data from another two nested case-control studies showed no significant association between Cr levels and the risk of GDM in pregnant women [15,42]. Studies exploring the relationship between Co exposure and GDM are limited; in a prospective cohort study, Co was shown to be significantly and positively associated with GDM [10]. The relationship between Se and GDM has been well established, and most studies support the negative association between Se and the risk of GDM [43]. A recent meta-analysis involving 27 studies showed that the serum Se level of patients with GDM was lower than that in healthy pregnant women [43].

In our present study, although no significant association was observed between Cr, Co, and Se concentrations and GDM in either a single-element or element coexposure logistic regression model. The plasma Cr and Se levels of patients with GDM were lower than those in the control group in our present study. In addition, the positive association between Co and GDM and the negative association between Se and GDM were consistent in the BKMR model and QGC analysis, and Co showed a positive non-linear relationship with FPI, OGTT-1h, and OGTT-2h in RCS analysis.

Our present results showed a much higher concentration of plasma Cr and Co and a comparable concentration of Se than those of previously published studies. Several previous studies showed that the median values varied from 0.2 to 3.97 μg/L for Cr [15,39,44], 1.68–1.9 mg/dL for Co [41,45], and 29.43–94.73 μg/L for Se [15,41,46]. The discrepancies between study populations remain to be elucidated because, aside from Cd, Cr and Co were reported to be the greatest heavy metal pollutant (Cr > Cd > Co > Zn > Ti > Cu) in the surface sediments of the Yangtze River Estuary [47], and there may indeed be a much higher level of metal/element exposure in the Shanghai population. In addition, Cr (III) and Se have been considered to have nutritional or pharmacological effects on the human body [48,49], ranging from antioxidant and anti-inflammatory effects to improving symptoms of insulin resistance, and Se is part of, for instance, Novalac Prenatal pills. The higher plasma level of Se and Cr in pregnant women in the non-GDM group may be the result of their using supplements containing Cr and Se before or during pregnancy. Further well-designed studies should be carried out to explore the role of Cr, Co, and Se in the occurrence, development, and treatment of GDM.

We adopted the BKMR method in our study to determine the joint effects of elements, but the results showed that the increasing percentile of element mixtures was not related to an increased risk of GDM. We speculate that the main explanation is that the contributing effect and protective effect of these six metallic elements on GDM offset each other, as shown in the QGC analysis. Notably, the association between Ni with GDM and Mn with GDM becomes statistically significant along with the increasing percentile of other elements in the BKMR model. There may exist a relatively strong interaction between Ni, Mn, and other elements.

Our study has several strengths. First, we collected blood samples during the first period of pregnancy, which may reflect the causal relationship between element exposure and the risk of GDM. Second, the exposure levels of metallic elements were reflected by a continuous variable (Ln-concentration), which avoids the data loss caused by classification variable conversion. Third, we used several statistical methods to assess the joint effect of all six elements and the independent contribution of each element on the risk of GDM, and the results were stable among these models.

Limitations should also be considered. First, detailed information regarding other potential confounding factors of GDM, such as physical activity and the occupational status during pregnancy, was not well collected. Second, plasma is not the best biological material to reflect body exposure to some elements, such as Ni. Third, we did not take exposure sources such as dietary patterns and residential environment into consideration.

## 5. Conclusions

In conclusion, our results suggest a positive association between V exposure and a negative association between Ni exposure in early pregnancy with subsequent risk of GDM, regardless of whether they are evaluated individually or as elements mixtures. Plasma Mn was found to be positively associated with an increased risk of GDM in the multiple-element model. In addition, we demonstrate a J-shaped exposure-response relationship between plasma Ni concentrations and GDM and a U-shaped exposure-response relationships between plasma Ni concentrations and FPI, FPG, OGTT-1h, and OGTT-2h. Further studies are warranted to confirm these associations and explore the potential mechanism.

## Figures and Tables

**Table 1:** Characteristics of the study population.

Characteristic	Total (*n* = 1166)	Non-GDM (*n* = 763)	GDM (*n* = 403)	*p*
Maternal age (years)	32.00 (30.00–34.00)	32.00 (30.00–34.00)	32.00 (30.00–34.00)	0.820
Pre-pregnancy BMI (kg/m^2^)				
Underweight (<18.5)	106 (9.10%)	67 (8.80%)	39 (9.70%)	0.056
Normal weight (18.5–23.9)	838 (71.90%)	565 (74.00%)	273 (67.70%)	
Overweight (≥24.0)	222 (19.00%)	131 (17.20%)	91 (22.60%)	
Education level (years)				
High school and lower	89 (7.63%)	55 (7.21%)	34 (8.40%)	0.037 *
Junior or college	240 (20.58%)	141 (18.48%)	99 (24.60%)	
University and higher	837 (71.78%)	567 (74.31%)	270 (67.00%)	
Household income (million Yuan)				
<0.1	88 (7.50%)	64 (8.40%)	24 (5.96%)	0.438
0.2–0.3	739 (63.38%)	483 (63.30%)	256 (63.52%)	
>0.3	339 (29.10%)	216 (28.30%)	123 (30.52%)	
Ethnic groups				
Ethnic Han	1150 (98.60%)	756 (99.10%)	394 (97.80%)	0.060
Others	16 (1.40%)	7 (0.90%)	9 (2.20%)	
Family history of diabetes (Yes)	146 (12.50%)	85 (11.10%)	61 (15.10%)	0.133
Smoking (Yes)	3 (0.30%)	3 (0.40%)	0 (0.00%)	0.277
Drinking (Yes)	6 (0.50%)	4 (0.50%)	2 (0.50%)	0.987
Parity				
Nulliparous	834 (71.53%)	550 (72.08%)	284 (70.47%)	0.728
Multiparous	332 (28.47%)	213 (27.92%)	119 (29.53%)	
Cesarean section (Yes)	554 (51.30%)	358 (50.90%)	196 (52.10%)	0.690
Infant sex				
Male	574 (49.20%)	377 (49.41%)	197 (48.88%)	0.716
Female	506 (43.40%)	327 (42.86%)	179 (44.42%)	
Missing	86 (7.40%)	59 (7.73%)	27 (6.70%)	

GDM, gestational diabetes mellitus; BMI, body mass index; * *p* < 0.05.

**Table 2:** Profiling of trace elements in maternal plasma of the case-control group.

Element	Total (*n* = 1166)	Non-GDM (*n* = 763)	GDM (*n* = 403)	*p*
V (μg/L)	6.25 (3.71–9.06)	6.02 (3.32–8.91)	6.60 (4.23–9.18)	0.007 **
Cr (μg/L)	372.40 (250.75–531.44)	391.38 (253.32–535.58)	342.77 (239.58–517.50)	0.021 *
Mn (μg/L)	5.79 (3.51–8.90)	5.61 (3.28–8.88)	5.91 (3.81–9.04)	0.076
Co (μg/L)	56.82 (40.24–81.94)	56.76 (39.66–81.81)	57.02 (41.23–82.13)	0.289
Ni (μg/L)	30.67 (17.34–48.57)	30.84 (18.69–48.89)	30.23 (15.10–48.40)	0.110
Se (μg/L)	87.80 (60.33–120.72)	89.76 (62.16–122.95)	84.30 (58.89–112.91)	0.044 *

GDM, gestational diabetes mellitus; V, Vanadium; Cr, Chromium; Mn, Manganese; Co, Cobalt; Ni, Nickel; Se, Selenium; * *p* < 0.05, ** *p* < 0.01.

**Table 3:** Associations between plasma trace element exposure and GDM risk.

Ln-Elements	Single-Element Model OR (95% CI)				Multi-Element Model OR (95% CI)	
	Modle 1	Modle 2	Modle 3	Modle 4	Crude Model	Adjusted Model
V (μg/L)	1.39 (1.14, 1.69) ***	1.47 (1.20, 1.82) ***	1.48 (1.20, 1.82) ***	1.47(1.20, 1.80) ***	1.27 (1.01, 1.60) *	1.37 (1.07, 1.76) *
Cr (μg/L)	0.81 (0.63, 1.03)	0.79 (0.6, 1.02)	0.77 (0.59, 1.00)	0.78 (0.60, 1.02)	0.82 (0.53, 1.27)	0.75 (0.47, 1.20)
Mn (μg/L)	1.22 (0.99, 1.51)	1.20 (0.96, 1.49)	1.21 (0.97, 1.51)	1.20 (0.96, 1.49)	1.70 (1.22, 2.36) **	1.83 (1.30, 2.59) ***
Co (μg/L)	1.14 (0.91, 1.43)	1.13 (0.89, 1.44)	1.14 (0.89, 1.46)	1.13 (0.89, 1.45)	1.32 (0.95, 1.85)	1.35 (0.94, 1.93)
Ni (μg/L)	0.86 (0.77, 0.97) *	0.82 (0.73, 0.93) **	0.82 (0.72, 0.93) **	0.82 (0.72, 0.93) **	0.72 (0.60, 0.86) ***	0.66 (0.54, 0.80) ***
Se (μg/L)	0.85 (0.67, 1.07)	0.86 (0.67, 1.10)	0.84 (0.66, 1.07)	0.86 (0.67, 1.10)	0.82 (0.57, 1.20)	0.87 (0.59, 1.27)

GDM, gestational diabetes mellitus; OR, odds ratio; CI, confidence interval; * *p* < 0.05, ** *p* < 0.01, *** *p* < 0.001. Model 1 adjusted by education level, ethnic groups, TG, LDL cholesterol, HDL cholesterol, and APOB. Model 2 adjusted by family history of diabetes, education level, ethnic group, TG, LDL cholesterol, and APOB. Model 3 adjusted by age, pre-pregnancy BMI, family history of diabetes, education level, ethnic group, household income level, TG, CHOL, LD, HDL cholesterol, and APOB. Adjusted model adjusted by family history of diabetes, education level, ethnic groups, household income level, TG, LDL cholesterol, HDL cholesterol, and APOB.

## Data Availability

The data presented in this study are available on reasonable request from the corresponding author.

## Supplementary Materials

The following supporting information can be downloaded at: https://www.mdpi.com/article/10.3390/nu15010115/s1, Figure S1: Correlations of the plasma concentration of the six trace elements. Spearman’s rank correlation was used to analyze the correlations between the Ln-transformed trace elements. Correlation coefficients are presented in the lower left part, and the upper right part is the heatmap of the correlation coefficients between Ln-transformedtrace elements. Blue represents a positive correlation, and the darker the color is, the greater the correlation coefficient. * *p* < 0.05, ** *p* < 0.01, *** *p* < 0.001. Figure S2: The dose-response relationships of plasma Ni with FPI and OGTT values. The blue line and gray shadow represent the OR value and 95% CI, respectively. (a) The dose-response relationship of Ln-Ni with FPI. (b) The dose-response relationship of Ln-Ni with FPG. (c) The dose-response relationship of Ln-Ni with OGTT-1h. (d) The dose-response relationship of Ln-Ni with OGTT-2h. Figure S3: The dose-response relationships of plasma V with FPI and OGTT values. The blue line and gray shadow represent the OR value and 95% CI, respectively. (a) The dose-response relationship of Ln-V with FPI. (b) The dose-response relationship of Ln-V with FPG. (c) The dose-response relationship of Ln-V with OGTT-1h. (d) The dose-response relationship of Ln-V with OGTT-2h. Figure S4: The dose-response relationships of plasma Mn with FPI and OGTT values. The blue line and gray shadow represent the OR value and 95% CI, respectively. (a) The dose-response relationship of Ln-Mn with FPI. (b) The dose-response relationship of Ln-Mn with FPG. (c) The dose-response relationship of Ln-Mn with OGTT-1h. (d) The dose-response relationship of Ln-Mn with OGTT-2h. Figure S5: The dose-response relationships of plasma Co with FPI and OGTT values. The blue line and gray shadow represent the OR value and 95% CI, respectively. (a) The dose-response relationship of Ln-Co with FPI. (b) The dose-response relationship of Ln-Co with FPG. (c) The dose-response relationship of Ln-Co with OGTT-1h. (d) The dose-response relationship of Ln-Co with OGTT-2h. Figure S6: The dose-response relationships of plasma Cr with FPI and OGTT values. The blue line and gray shadow represent the OR value and 95% CI, respectively. (a) The dose-response relationship of Ln-Cr with FPI. (b) The dose-response relationship of Ln-Cr with FPG. (c) The dose-response relationship of Ln-Cr with OGTT-1h. (d) The dose-response relationship of Ln-Cr with OGTT-2h. Figure S7: The dose-response relationships of plasma Se with FPI and OGTT values. The blue line and gray shadow represent the OR value and 95% CI, respectively. (a) The dose-response relationship of Ln-Se with FPI. (b) The dose-response relationship of Ln-Se with FPG. (c) The dose-response relationship of Ln-Se with OGTT-1h. (d) The dose-response relationship of Ln-Se with OGTT-2h. Figure S8: LASSO regression analysis diagram. Maternal age, pre-BMI, ethnic group, educational level, household income level, family history of diabetes, TG, CHOL, LDL, HDL, APO-B, and 6 trace elements were included in the LASSO regression model for analysis. (a) The changing trajectory of misclassification error with the penalty parameter (logλ) (estimates and 95% CI). The dotted line represents the optimal λ value selected after cross-validation. (b) Cross-validation plot for the penalty term. Supplementary Figure S9: Effect of single trace element exposure on GDM by BKMR model Univariate exposure-response functions of each element (95% CI) with others fixed at their medians (P50). The results were adjusted by family history of diabetes, education level, ethnic groups, TG, LDL-cholesterol, and APO-B. Figure S10: Bivariate cross-section effects of trace element exposure on GDM by BKMR model Bivariate cross-section effects of the exposure-response function of a single element where the second element was fixed at P25, P50, and P75. Table S1: Profiling of trace elements in maternal plasma (*n* = 1166) Table S2: Level of glucose and lipid metabolism indices.
