# Supplementary material for: Association between Plasma Trace Element Concentrations in Early Pregnancy and Gestational Diabetes Mellitus in Shanghai, China
Source: Nutrients. 2022 Dec 27;15(1):115. doi: 10.3390/nu15010115 (PMC9824253; doi:10.3390/nu15010115)
Supplement: Supplementary file 1 [file nutrients-15-00115-s001.zip › Supplementart Table1-2.pdf]

Association between plasma trace elements concentrations in early pregnancy and gestational diabetes mellitus in Shanghai, China

Ting Wu1,2 †, Tao Li1,2 †, Chen Zhang1,2, Hefeng Huang1,2,3,4,5**, Yanting Wu3,4*
Affiliations:
1 The International Peace Maternity and Child Health Hospital, School of Medicine, Shanghai Jiao Tong University, Shanghai 200030, China
2 Shanghai Key Laboratory of Embryo Original Diseases, Shanghai 200030, China
3Obstetrics and Gynecology Hospital, Institute of Reproduction and Development, Fudan University, Shanghai, China.
4Research Units of Embryo Original Diseases, Chinese Academy of Medical Sciences, Shanghai 200030, China
5 Women's Hospital, School of Medicine, The Key Laboratory of Reproductive Genetics, Ministry of Education (Zhejiang University), Hangzhou 310058, China
† Ting Wu and Tao Li contributed equally to this work.

*** Correspondence:**
Yan-Ting Wu,
Obstetrics and Gynecology Hospital, Fudan University, No. 419, Fangxie Rd., Shanghai, 200000, China,
E-mail: yanting_wu@163.com,
Tel: +86-21-33189900;
ORCID: 0000-0002-2293-1792

He-Feng Huang,
Obstetrics and Gynecology Hospital, Fudan University, No. 419, Fangxie Rd., Shanghai, 200000, China,
Email: huanghefg@sjtu.edu.cn ,
Tel: 86-21-64070434,
ORCID: 0000-0003-4344-8019

TableS1.Profiling of trace elements in maternal plasma(n=1166)

TableS2.Level of glucose and lipid metabolism indices

TableS1. Profiling of trace elements in maternal plasma (n= 1166)

Elements (μg/L)	LOD	Percent of detection	Mean	Minimum	Percentile			Maximum
					25th	50th	75th	
Vanadium	0.0119	1157 (99.23%)	7.44	0.169	3.71	6.25	9.06	112.81
Chromium	0.3059	1166 (100%)	407.27	34.301	250.75	372.40	531.44	1217.98
Manganese	0.0076	1131 (97.00%)	6.90	0.587	3.51	5.79	8.90	127.69
Cobalt	0.0125	1166 (100%)	64.84	0.177	40.24	56.82	81.94	301.02
Nickle	0.0092	1021 (87.56%)	38.80	2.641	17.34	30.67	48.58	1019.21
Selenium	0.3063	1161 (99.57%)	96.96	4.333	60.33	87.80	120.72	324.82

LOD: Limit of detection

TableS2. Level of glucose and lipid metabolism indices

	Total (n= 1166)	Non-GDM (n= 763)	GDM (n= 403)	*p*
FPG (mmol/L)	4.67 (4.38-5.04)	4.49 (4.27-4.71)	5.19 (4.89-5.45)	<0.001***
OGTT-1h	8.11 (7.12-9.51)	7.70 (6.64-8.45)	10.04 (8.52-10.84)	<0.001***
OGTT-2h	6.79 (5.99-7.80)	6.45 (5.71-7.09)	8.10 (6.93-8.97)	<0.001***
FPI (μU/mL)	6.70 (4.80-8.90)	6.40 (4.50-8.30)	7.30 (5.60-9.90)	<0.001***
HOMA-IR	1.42 (0.98-1.92)	1.26 (0.88-1.69)	1.69 (1.30-2.31)	<0.001***
HbA1c, %	5.30 (5.10-5.43)	5.30 (5.10-5.40)	5.30 (5.20-5.50)	<0.001***
CHOL (mmol/L)	4.46 (3.98-4.96)	4.40 (3.93-4.93)	4.53 (4.10-4.99)	<0.001***
TG (mmol/L)	1.31 (1.06-1.64)	1.26 (1.02-1.60)	1.41 (1.14-1.78)	0.003**
LDL cholesterol (mmol/L)	2.47 (2.09-2.86)	2.43 (2.04-2.82)	2.54 (2.16-2.95)	<0.001***
HDL cholesterol (mmol/L)	1.89 (1.65-2.16)	1.90 (1.67-2.20)	1.87 (1.62-2.11)	0.008**
APO-A (mmol/L)	1.77 (1.68-1.87)	1.77 (1.67-1.87)	1.79 (1.69-1.87)	0.116
APO-B (mmol/L)	0.80 (0.71-0.90)	0.79 (0.70-0.89)	0.82 (0.73-0.90)	0.001**

FPG: fasting plasma glucose; OGTT, oral glucose tolerance test; FPI: fasting plasma insulin; HOMA-IR, homeostasis model of assessment-insulin resistance; HbA1c: glycosylated Hemoglobin; *P < 0.05, ** P < 0.01, ***P < 0.001.
