# Supplementary figures and images for: Association between Plasma Trace Element Concentrations in Early Pregnancy and Gestational Diabetes Mellitus in Shanghai, China
Source: Nutrients. 2022 Dec 27;15(1):115. doi: 10.3390/nu15010115 (PMC9824253; doi:10.3390/nu15010115)

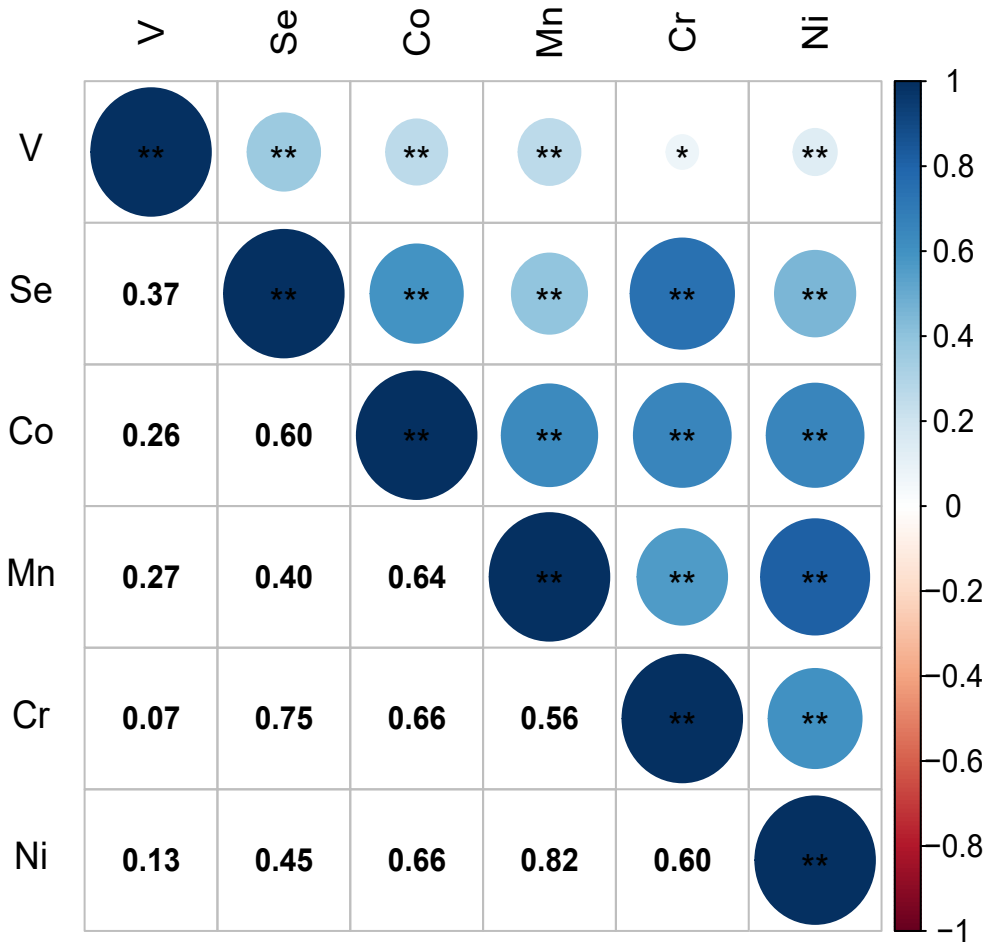

Supplement: Supplementary file 1 [file nutrients-15-00115-s001.zip › FigureS1.pdf]

$h(\text{expos1} - \text{quantile of expos2})$

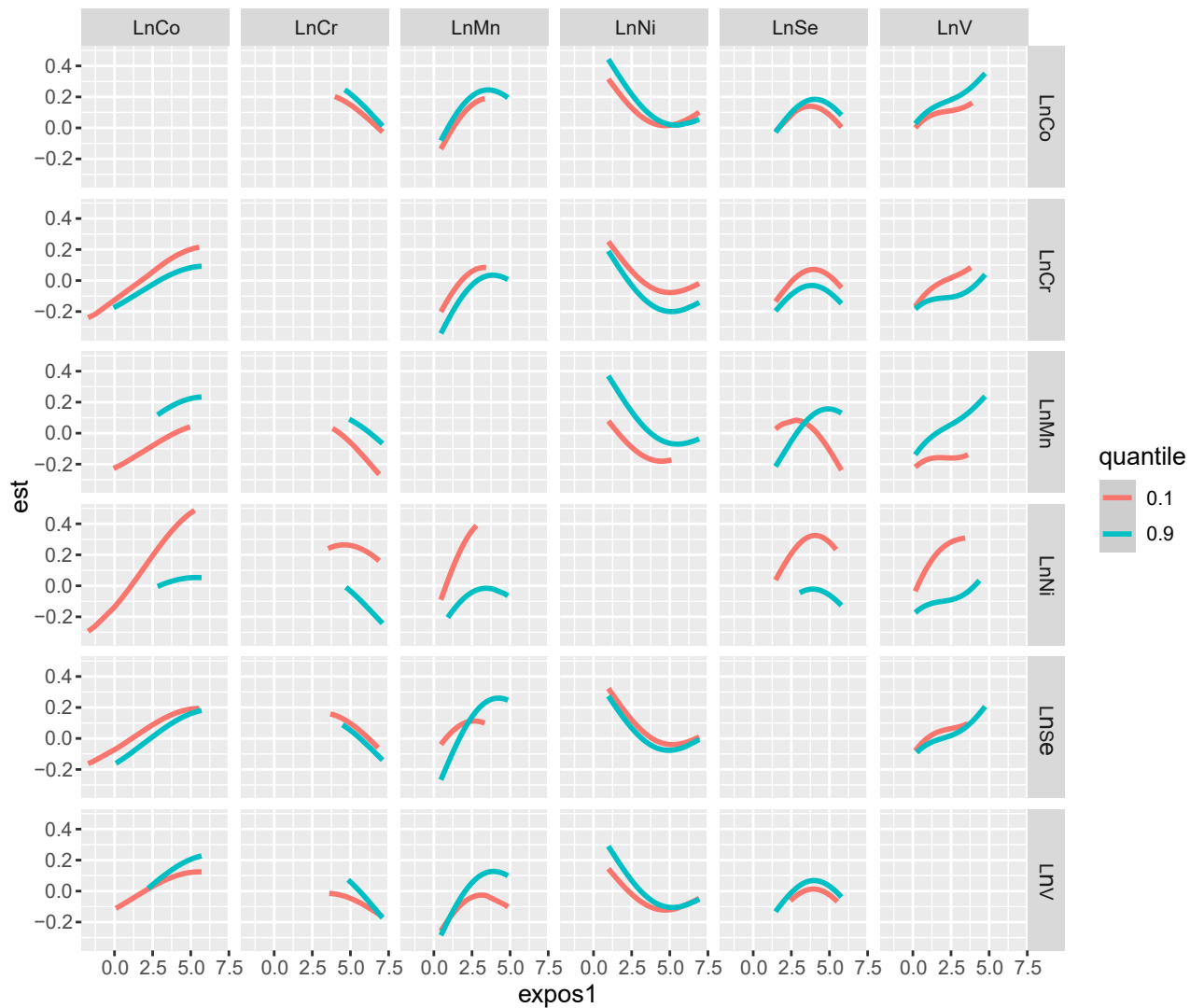

Supplement: Supplementary file 1 [file nutrients-15-00115-s001.zip › FigureS10.pdf]

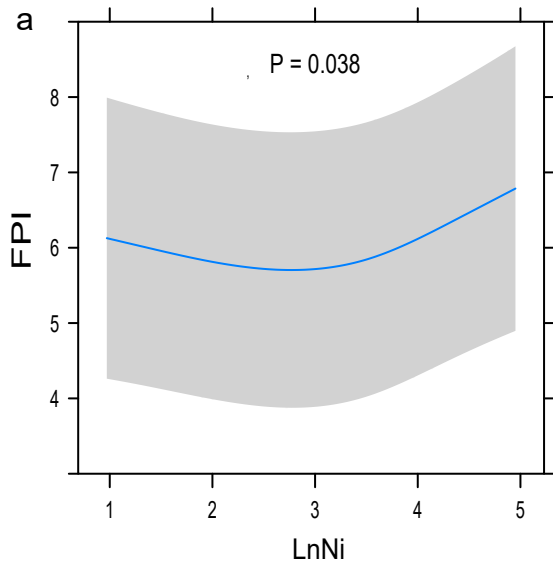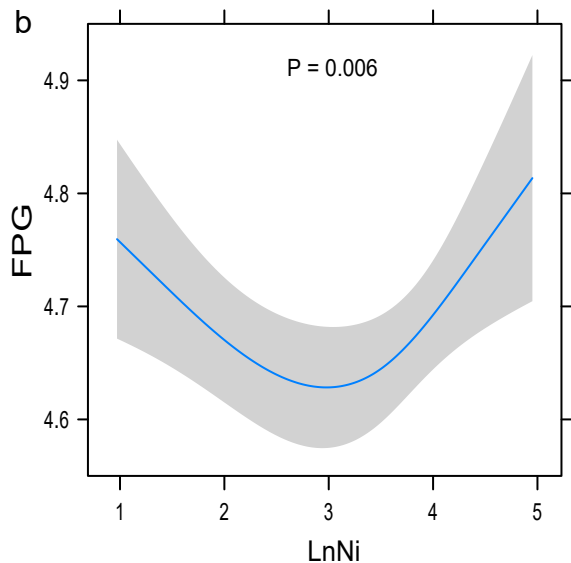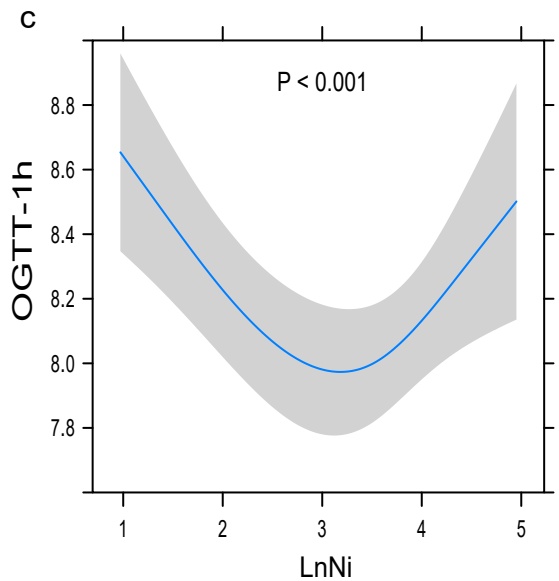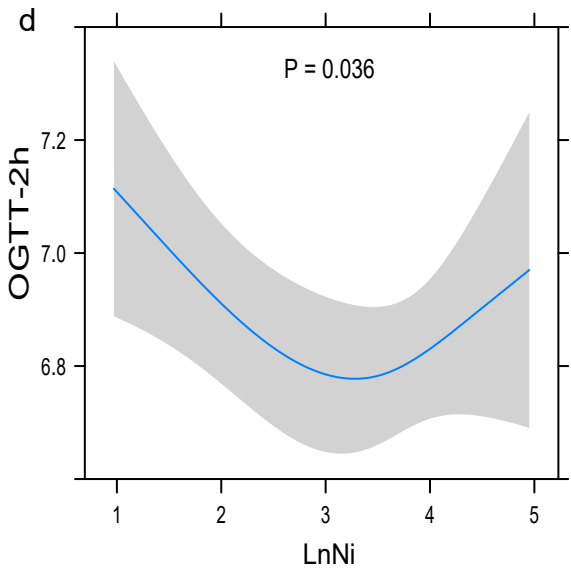

Supplement: Supplementary file 1 [file nutrients-15-00115-s001.zip › FigureS2.pdf]

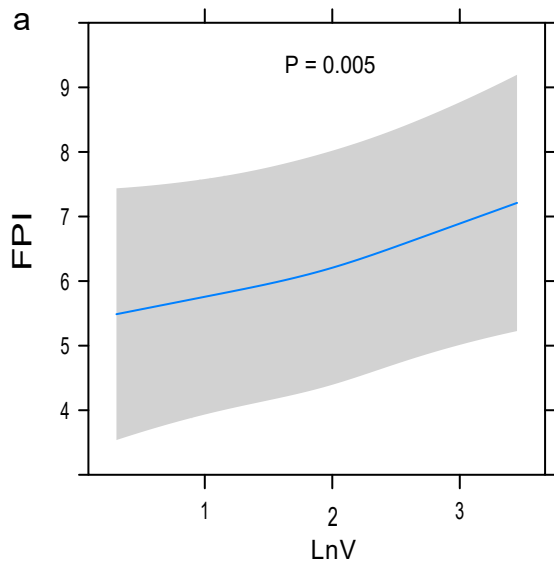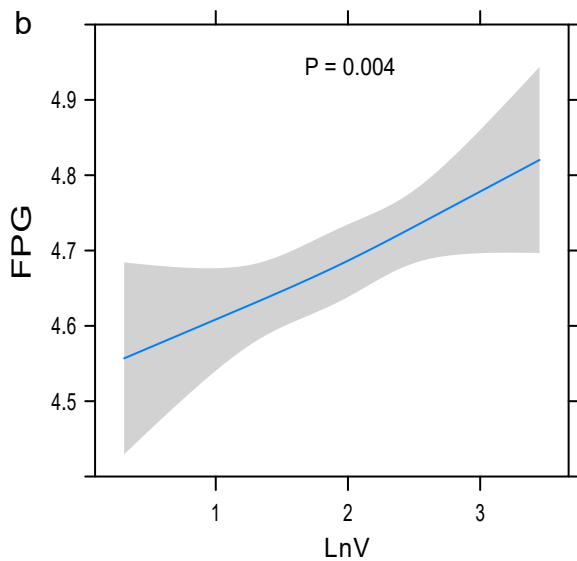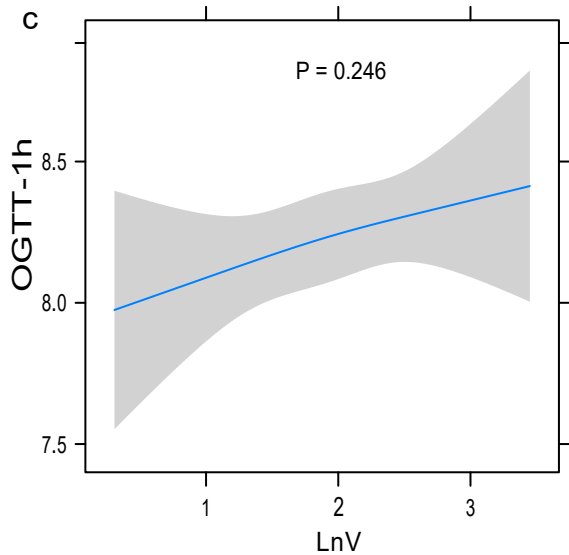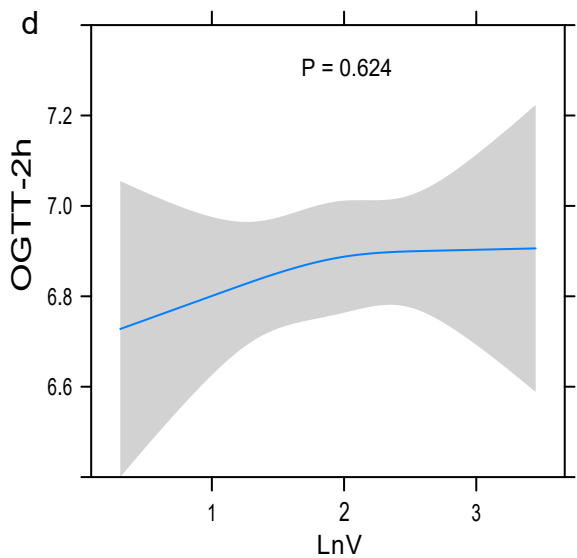

Supplement: Supplementary file 1 [file nutrients-15-00115-s001.zip › FigureS3.pdf]

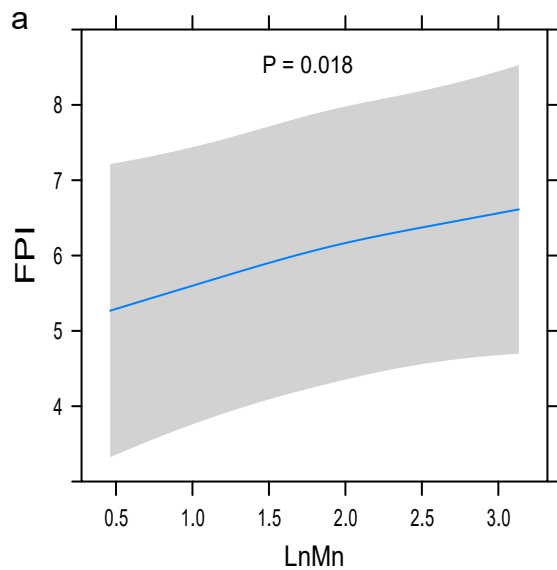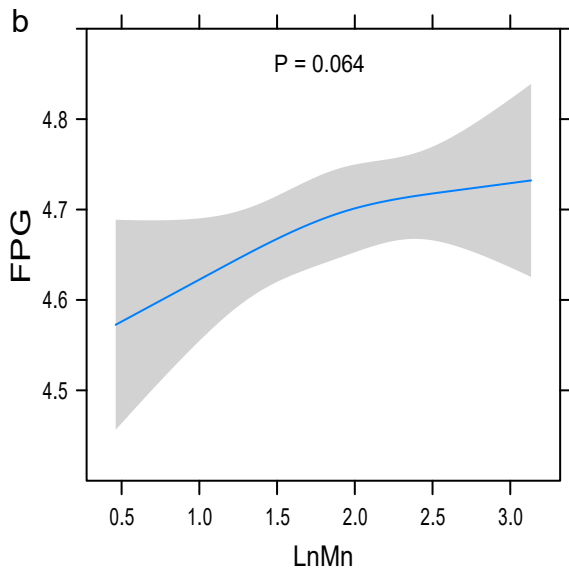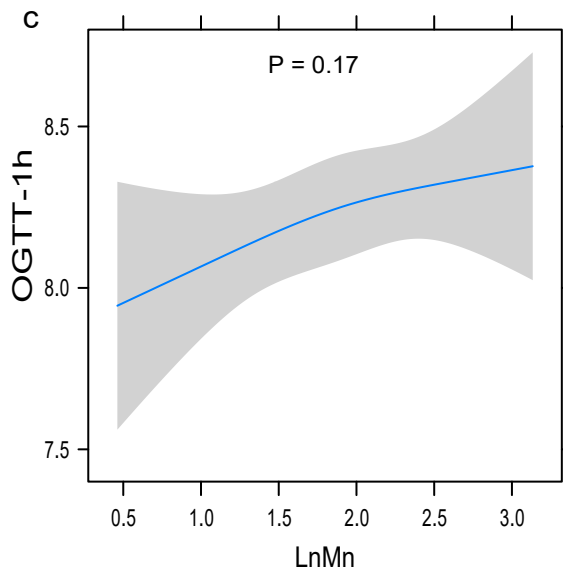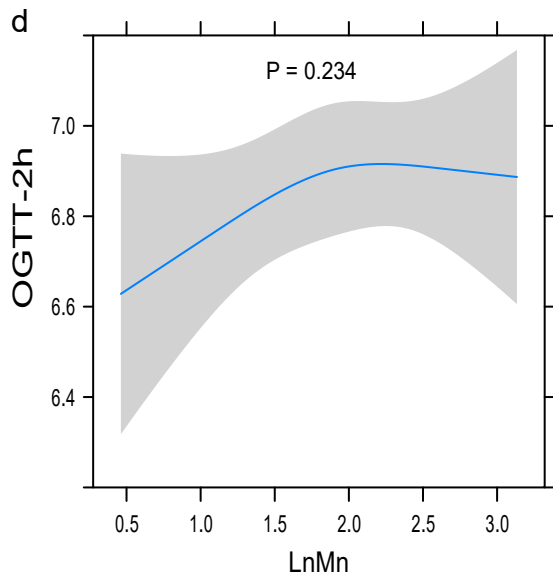

Supplement: Supplementary file 1 [file nutrients-15-00115-s001.zip › FigureS4.pdf]

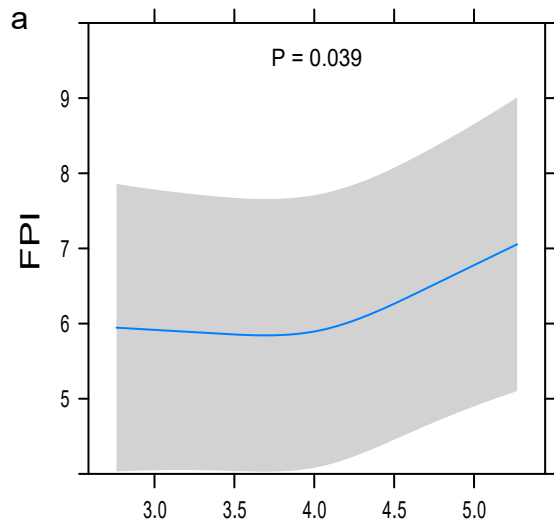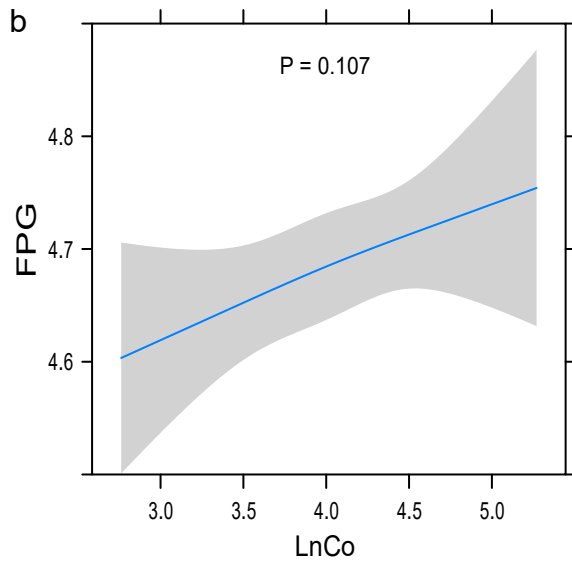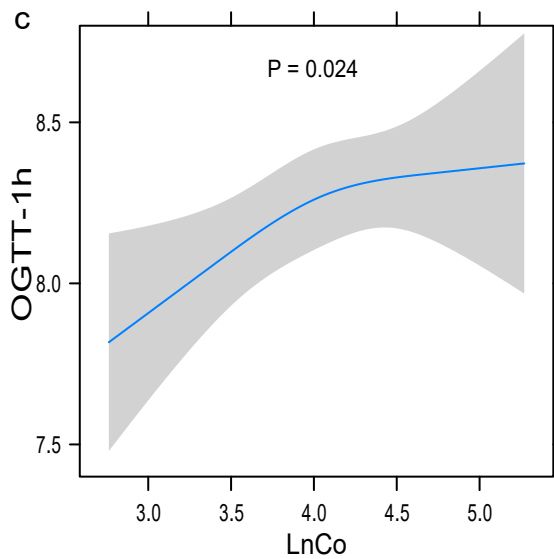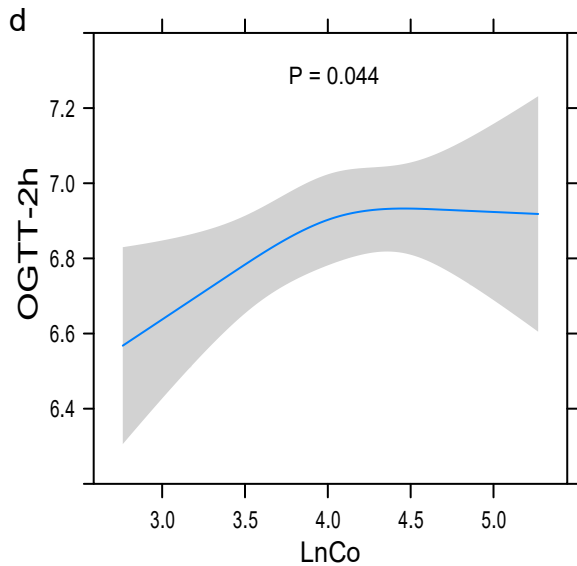

Supplement: Supplementary file 1 [file nutrients-15-00115-s001.zip › FigureS5.pdf]

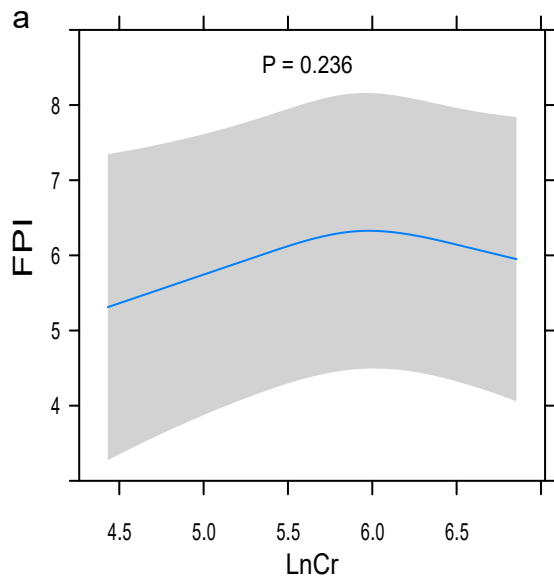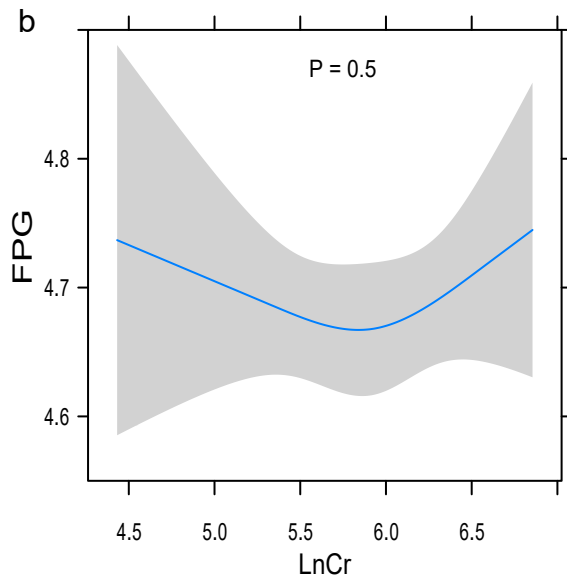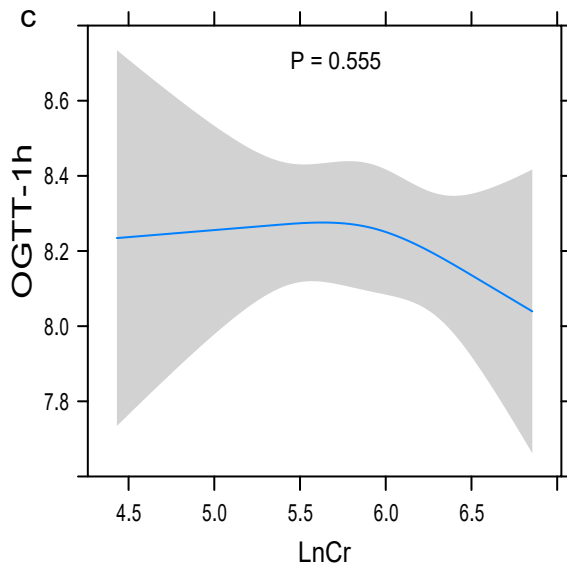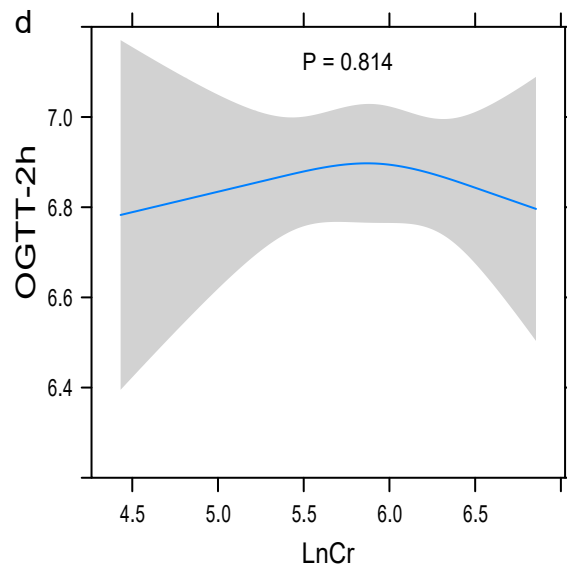

Supplement: Supplementary file 1 [file nutrients-15-00115-s001.zip › FigureS6.pdf]

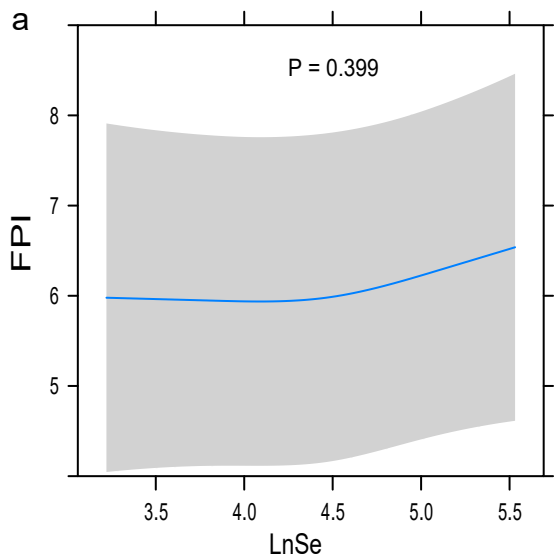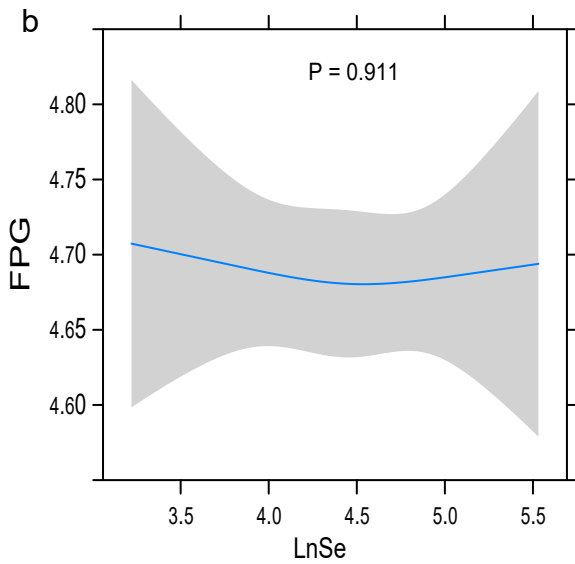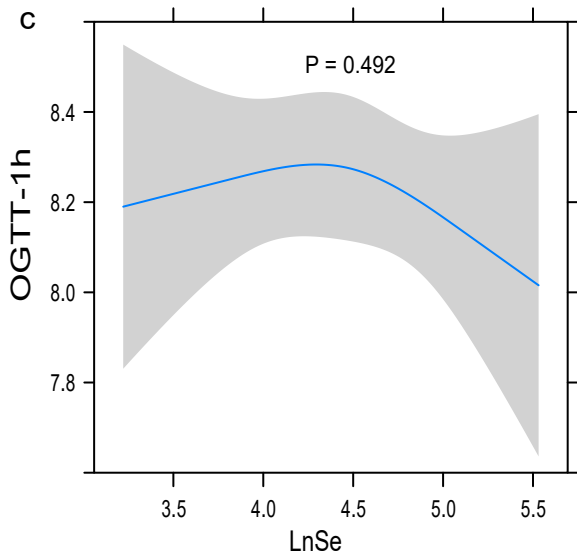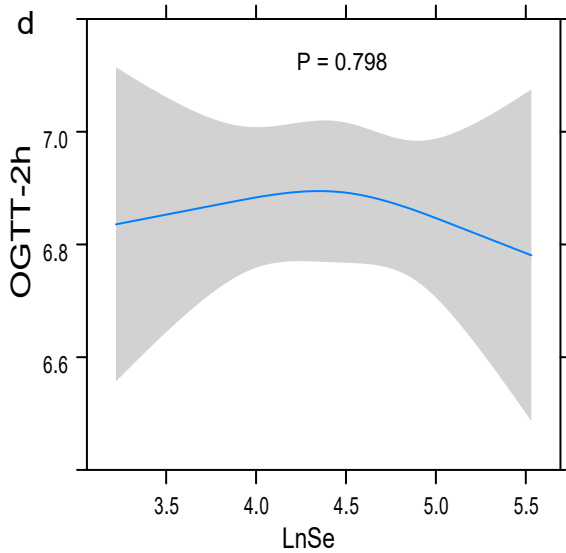

Supplement: Supplementary file 1 [file nutrients-15-00115-s001.zip › FigureS7.pdf]

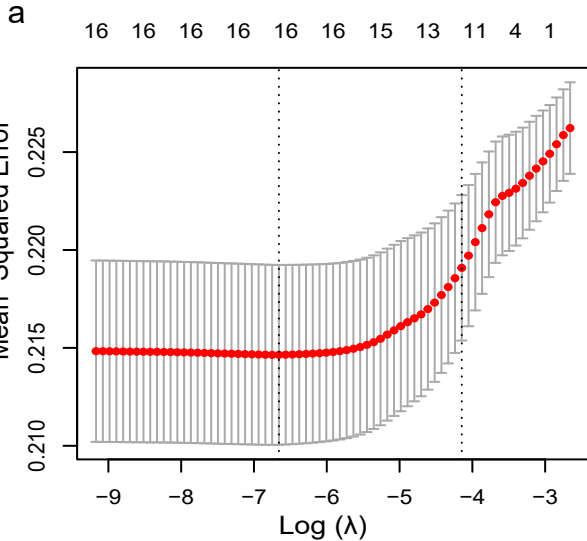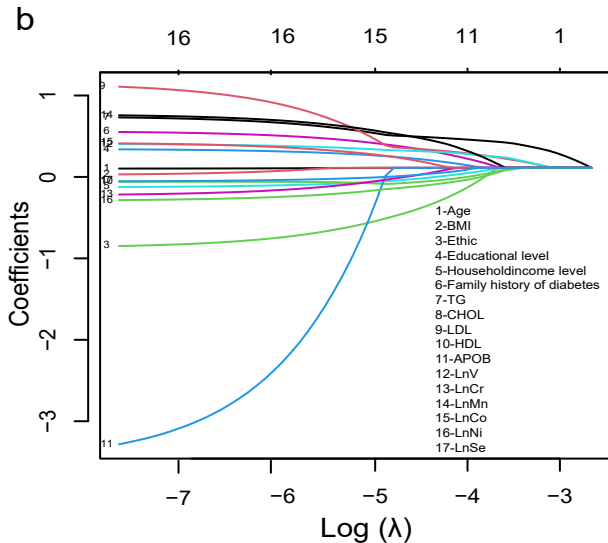

Supplement: Supplementary file 1 [file nutrients-15-00115-s001.zip › FigureS8.pdf]

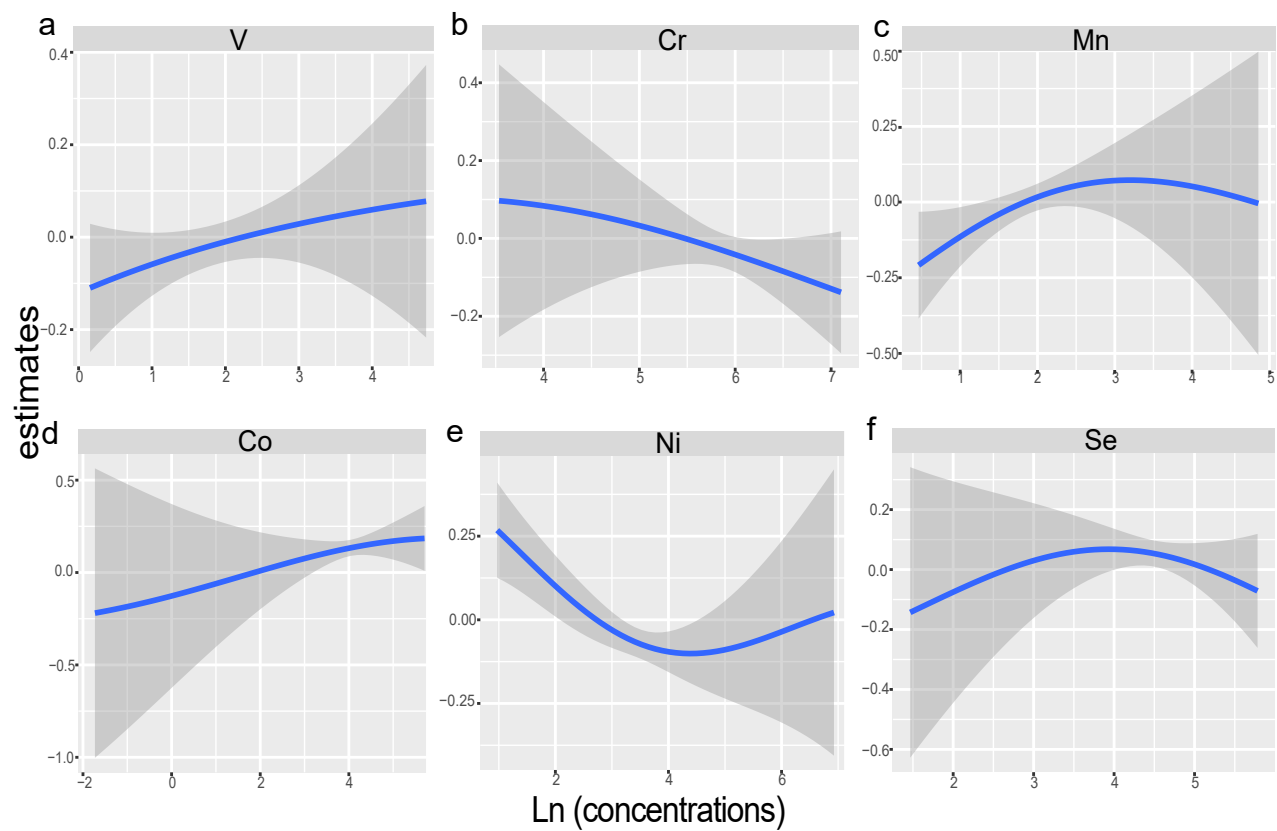

Supplement: Supplementary file 1 [file nutrients-15-00115-s001.zip › FigureS9.pdf]
